# Comparing Holotranscobalamin and Total Vitamin B12 in Diagnosing Vitamin B12 Deficiency in Megaloblastic Anemia Patients

**DOI:** 10.7759/cureus.71278

**Published:** 2024-10-11

**Authors:** Noareen Tufail, Muzammil Kataria, Ahmed Jamal Chaudhary, Rubaid A Dhillon, Muhammad Asif Naveed, Shahida Mohsin

**Affiliations:** 1 Haematology, University of Health Sciences, Lahore, PAK; 2 Internal Medicine, Jinnah Hospital, Lahore, PAK; 3 Internal Medicine, Detroit Medical Center (DMC) Sinai-Grace Hospital, Detroit, USA; 4 Internal Medicine, Riphah International University, Rawalpindi, PAK; 5 Pathology, University of Health Sciences, Lahore, PAK

**Keywords:** diagnostic accuracy, holotranscobalamin, megaloblastic anemia, methylmalonic acid, vitamin b12

## Abstract

Background

Megaloblastic anemia is characterized by abnormally large red blood cells caused by a deficiency in either vitamin B12 or folic acid, both of which are essential for DNA synthesis. Vitamin B12 insufficiency can lead to severe neurological damage, making early identification of vitamin B12 deficiency crucial to prevent irreversible harm. Vitamin B12 deficiency results in decreased levels of holotranscobalamin (Holo-TC) and increased levels of methylmalonic acid (MMA). Methylmalonic acid is considered the gold standard for diagnosing B12 deficiency because it is a specific marker that rises when B12 is insufficient, even when serum B12 levels appear normal. Elevated MMA levels reflect impaired B12 metabolism, making it a critical tool for early detection and intervention. Previous research indicates that Holo-TC, the active form of vitamin B12 available to cells, is a more specific diagnostic tool for early vitamin B12 deficiency than total B12. This study aims to determine the diagnostic validity of total vitamin B12 and Holo-TC using MMA as the gold standard in patients with megaloblastic anemia.

Methods

A total of 95 megaloblastic anemia patients were selected from Jinnah Hospital and Lahore General Hospital, Lahore, Pakistan, after receiving approval from the ethical review committees. This was a cross-sectional study. Whole blood, serum, and urine samples were collected in ethylenediamine tetraacetic acid (EDTA) vials, gel vials, and urine containers, respectively. The EDTA samples were used for complete blood count measurements using a hematology analyzer (Sysmex-XT 1800i, Sysmex America, Inc., Mundelein, IL), while serum and urine samples were employed for the detection of serum folic acid, cobalamin, Holo-TC, and MMA levels through manual enzyme-linked immunosorbent assay (ELISA) techniques. Sensitivity, specificity, positive predictive value (PPV), and negative predictive value (NPV) of Holo-TC and cobalamin were calculated.

Results

The majority of patients fell within the age group of 20-70 years, with 57% of them being females and 43% males; Holo-TC exhibited a sensitivity of 98.9% and specificity of 50.00%, with a PPV of 98.90% and NPV of 50%. Vitamin B12 demonstrated a sensitivity of 63% and specificity of 50%, with a PPV of 98.33% and NPV of 2.85%. The diagnostic accuracy of Holo-TC and vitamin B12 was observed to be 97.8% and 63%, respectively.

Conclusions

Between the two, Holo-TC displays higher diagnostic accuracy than vitamin B12 and can serve as the primary test for patients suspected of having vitamin B12 deficiency.

## Introduction

Megaloblastic anemia comprises a group of disorders resulting from defective DNA synthesis and nucleus-cytoplasmic asynchrony, leading to anemia characterized by abnormally large red blood cells [1]. This condition can cause a variety of symptoms due to the impaired production of red blood cells. Both vitamin B12 and folate are crucial for DNA synthesis, and a deficiency in either or both of these vitamins can cause megaloblastic anemia [2]. Among these, vitamin B12 deficiency stands out as a major leading cause of megaloblastic anemia. Inadequate dietary intake, overcooking of food, and poor absorption can contribute to vitamin B12 deficiency, leading to a higher incidence of the condition in populations with these risk factors. The incidence of megaloblastic anemia increases in countries with prevalent malnutrition, where routine vitamin supplementation is not available for vulnerable groups such as the elderly and pregnant women [3]. The presentation of megaloblastic anemia can vary among individuals, but common symptoms include fatigue, weakness, shortness of breath, loss of appetite, weight loss, fast heartbeat, a smooth or tender tongue, numbness and tingling in extremities, ataxia, and cognitive impairment [4]. These symptoms result from the body's inability to produce enough healthy red blood cells, which impairs oxygen delivery to tissues.

In Pakistan, megaloblastic anemia is frequently encountered in clinical practice, and vitamin B12 deficiency is common in the Pakistani population. For example, a study observed vitamin B12 deficiency in 72.6% of the study population [5]. Another study by Naeem and Uttra (2007) found vitamin B12 deficiency in 31.8% of the population, while a study by Nizamani et al. (2014) reported the prevalence of B12 deficiency at 57% [6, 7]. A study by Devrajani et al. (2011) observed a prevalence of vitamin B12 deficiency at 48%. These statistics underscore the significant burden of B12 deficiency in Pakistan [8].

Methylmalonic acid (MMA) serves as a specific marker of vitamin B12 deficiency [9]. Both MMA and homocysteine levels increase in cases of vitamin B12, B6, and folate deficiency [8]. Previous literature has demonstrated that cobalamin and folate deficiency can be differentiated by measuring MMA and homocysteine levels. Homocysteine levels are elevated in cases of folate deficiency, whereas MMA levels are predominantly elevated in cobalamin deficiency [10]. This differentiation is important for accurate diagnosis and treatment.

Holotranscobalamin (Holo-TC), an active fraction of cobalamin, serves as a sensitive marker with a sensitivity of 93.33% and specificity of 80% [11]. It is superior and an early marker for detecting changes in vitamin B12 status within the body compared to total vitamin B12 levels [12]. Holotranscobalamin represents biologically available B12 since it is utilized by cells for various functions such as DNA synthesis, providing insight into early changes in B12 levels. Low serum Holo-TC levels indicate insufficient vitamin B12 stores. Low serum Holo-TC and high MMA levels indicate B12 deficiency [13]. This marker is particularly helpful in cases where there is no correlation between vitamin B12 levels and its metabolites. Various suitable methods are now available to measure Holo-TC [14].

Therefore, it is essential to diagnose early or subclinical cases of cobalamin deficiency before irreversible neurological damage occurs. The present study aimed to evaluate cobalamin deficiency using newly established parameters like Holo-TC with MMA as the proxy gold standard. The results of these patients will also be compared with already established parameters like vitamin B12 levels. While total vitamin B12 levels may miss patients with vitamin B12 deficiency, Holo-TC appears to be a more sensitive marker, supporting its use as a primary parameter for detecting cobalamin deficiency. This approach could improve early diagnosis and treatment, preventing severe complications.

## Materials and methods

A cross-sectional study was conducted in the Department of Hematology, University of Health Sciences (UHS), Lahore, Pakistan, following the approval of the study synopsis by the Advanced Studies Research Board of the UHS (approval number: UHS/ERC/23/118). A sample size of 95 was determined using health studies software (version 2.0.21 WHO, Geneva, Switzerland) with a 6% margin of error, a 95% confidence level, and an anticipated sensitivity of Holo-TC of 90%. The participants were selected through non-probability/consecutive sampling.

Inclusion and exclusion criteria

The inclusion criteria comprised adult patients of either sex aged 20-70 years with hemoglobin levels of less than 12 g/dL for males and less than 10 g/dL for females and a mean corpuscular volume (MCV) greater than 100 fl along with hyper-segmented neutrophils. Patients who were already receiving vitamin B12 therapy, taking multivitamins, undergoing blood transfusion therapy, had a history or diagnosis of chronic illnesses such as hypothyroidism, cardiac diseases, gastrointestinal disorders, pulmonary tuberculosis, or liver disease, were experiencing folate deficiency, or had a history of alcohol intake were excluded from the study.

Sample collection and laboratory analyses

After obtaining written consent from the patients, sample data were collected. A total of five milliliters of venous blood was drawn from each patient for the determination of the complete blood count (CBC) and peripheral smear morphology examination. Additionally, serum levels of folate, vitamin B12, Holo-TC, and urine MMA were measured using the enzyme-linked immunosorbent assay (ELISA) technique. Urine samples were collected in urine sampling jars for the measurement of urinary MMA levels.

In the laboratory, CBCs were performed using Sysmex XT 1800i (Sysmex America, Inc., Mundelein, IL) on collected ethylenediaminetetraacetic acid (EDTA) samples, and peripheral smears were prepared and examined under a microscope. One aliquot was used for serum B12, a second for serum folate, and the third aliquot was used for serum Holo-TC. Urine samples were used for MMA level measurements. Serum folate, cobalamin, Holo-TC, and MMA levels were determined using the ELISA technique. Details regarding the reagents, including the specific brands and concentrations used, the equipment, such as the Sysmex XT-1800i hematology analyzer, and the ELISA kits employed for serum folate, vitamin B12, Holo-TC, and MMA measurements, have been included to enhance the reproducibility of the analysis.

Statistical analyses

All statistical analyses were performed using IBM SPSS Statistics software, version 20.0, Armonk, NY. Descriptive statistics were used to summarize the baseline characteristics of the study participants, including age and gender distribution. Continuous variables were presented as means ± standard deviations (SD), and categorical variables were presented as frequencies and percentages.

Sensitivity, specificity, positive predictive value (PPV), and negative predictive value (NPV) of Holo-TC and cobalamin were calculated with corresponding 95% confidence intervals (CIs). Comparative analyses were conducted using chi-square tests for categorical variables and independent t-tests for continuous variables.

A regression analysis was performed to assess the relationship between Holo-TC and MMA levels, as well as between vitamin B12 and MMA levels. The coefficients, standard errors, and p-values were calculated to determine the significance of these relationships. The level of significance was set at a p-value < 0.05, and all p-values were two-tailed. Diagnostic accuracy was calculated for vitamin B12 and Holo-TC with MMA as the proxy gold standard. The study aimed to determine the diagnostic validity of total vitamin B12 and Holo-TC in detecting B12 deficiency in patients with megaloblastic anemia, with MMA serving as the reference standard.

## Results

Out of the total 95 megaloblastic anemia patients, 54 (57%) were female and 41 (43%) were male, falling within the age group of 20-70 years.

To assess normal distribution, the Shapiro-Wilk test was applied, revealing that Hb, red blood cell (RBC) count, MCH, folate, and MMA levels exhibited a normal distribution with p-values ≥ 0.05. The mean values for Hb, RBC count, mean corpuscular hemoglobin (MCH), folate, and MMA were 7.4 ± 2.1 g/dL, 3.08 ± 0.7 × 10^6/µL, 28.4 ± 3.6 pg, 420.8 ± 168.2 ng/mL, and 50.9 ± 16.6 ng/mL, respectively.

In contrast, age, MCV, mean corpuscular hemoglobin concentration (MCHC), white blood cell (WBC) count, platelets, vitamin B12, and Holo-TC parameters did not follow a normal distribution with p-values ≤ 0.05. The median with interquartile range (IQR) levels for these parameters were as follows: age = 37 (24) years, MCV = 110 (12) fl, MCHC = 31 (5) g/dL, WBC count = 4.1 (3.1) × 10^3/µL, platelet count = 120 (60) × 10^3/µL, B12 level = 201 (496) ng/L, and Holo-TC level = 0.16 (2.06) ng/mL (Table 1).

**Table 1:** Data distribution of the study population Hb: hemoglobin; RBC: red blood cell; MCV: mean corpuscular volume; MCH: mean corpuscular hemoglobin; MCHC: mean corpuscular hemoglobin concentration; WBC: white blood cell; Holo-TC: holotranscobalamin; MMA: methylmalonic acid

Variables	Mean ± SD	Median (IQR)	P-value	Data distribution
Age (years)	38.9 ±13.8	37 (24)	0.000	Not normal
Hb (g/dl)	7.4 ± 2.1	7.5(3.4)	0.127	Normal
RBC count (X10^6^/ul)	3.08 ± 0.7	3.1(0.9)	0.342	Normal
MCV (fl)	109 ± 6.3	110 (12)	0.000	Not normal
MCH (pg)	28.4 ± 3.6	29(5)	0.057	Normal
MCHC (g/dl)	31.2 ± 2.9	31 (5)	0.027	Not normal
WBC count (X10^3^/µl)	5.0 ± 2.7	4.1 (3.1)	0.000	Not normal
Platelet count (X10^3^/l)	130 ± 72	120 (60)	0.000	Not normal
Vitamin B12 level (ng/L)	417 ± 500	201(496)	0.000	Not normal
Folate (ng/ml)	420.8 ± 168.2	460(256)	0.079	Normal
Holo-TC (ng/ml)	1.88 ±4.0	0.16 (2.06)	0.000	Not normal
MMA (ng/ml)	50.9 ± 16.6	50.7(20.43)	0.852	Normal

Oval macrocytosis and hyper-segmented neutrophils were present in the peripheral blood of all 95 (100%) patients. Folate levels were elevated in all 95 (100%) selected patients. Among the 95 megaloblastic patients, 33 (34.7%) had very low, 14 (15%) had low, 14 (15%) had borderline low, 30 (31%) had normal, and four (4.3%) had high levels of B12.

The diagnostic accuracy of serum Holo-TC and serum B12 was quantified by measuring sensitivity, specificity, PPV, and NPV (Table 2). The sensitivity, specificity, PPV, and NPV were calculated using MMA as the proxy gold standard. The sensitivity of Holo-TC and B12 was found to be 98.9% and 63%, respectively. Specificity for both Holo-TC and B12 was observed to be 50%. The PPV for Holo-TC and B12 was 98.91% and 98.33%, respectively. The NPV for serum Holo-TC and serum B12 was 50% and 2.85%, respectively. The diagnostic accuracy of serum Holo-TC was calculated to be 97.87%, while that of serum cobalamin was 63.15%.

**Table 2:** Diagnostic performance of holotranscobalamin and serum B12 having methylmalonic acid as gold standard Holo-TC: holotranscobalamin; PPV: positive predictive value; NPV: negative predictive value

Parameters	Sensitivity, %	Specificity, %	PPV,%	NPV,%	Diagnostic accuracy
Serum B12	63	50	98.33	2.85	63.15
Serum Holo-TC	98.9	50	98.91	50	97.87

A regression analysis was conducted to further investigate the relationship between B12, Holo-TC, and MMA levels (Table 3). The coefficient for B12 was found to be 0.04, suggesting a slight positive association with MMA levels; however, the borderline significance indicates minimal impact. The coefficient for Holo-TC was found to be -1.20, indicating a significant negative association with MMA levels. Higher Holo-TC levels are associated with lower MMA levels. This analysis supports the findings from Table 2 that Holo-TC is a more sensitive and reliable marker for B12 deficiency.

**Table 3:** Regression analysis of holotranscobalamin and vitamin B12 against methylmalonic acid levels Holo-TC: holotranscobalamin

Predictor variables	Coefficient (β)	Standard error (SE)	t-value	p-value	95% CI lower	95% CI upper
Constant	45.2	3.4	13.29	0.000	38.5	51.9
Vitamin B12 level (ng/L)	0.04	0.02	2.00	0.048	0.00	0.08
Holo-TC (ng/mL)	-1.20	0.58	-2.07	0.042	-2.35	-0.05

## Discussion

Megaloblastic anemia is a hematological disorder characterized by megaloblastic changes in the bone marrow due to a defect in DNA synthesis. These changes result in the production of abnormally large and immature red blood cells, which are less effective at transporting oxygen throughout the body. The condition arises primarily from deficiencies in vitamin B12 and folic acid, both of which are essential for DNA synthesis and red blood cell formation. Numerous studies have highlighted the substantial burden of micronutrient deficiency, with vitamin B12 and folic acid deficiencies being the most significant contributors to megaloblastic anemia [15]. This highlights the critical need for proper nutritional intake to prevent such deficiencies.

The current study utilized MMA as the gold standard to evaluate the diagnostic validity of total vitamin B12 and Holo-TC in patients with megaloblastic anemia. Methylmalonic acid is a specific marker for vitamin B12 deficiency; its levels increase when vitamin B12 is insufficient.

Our study comprised 57.0% females, predominantly in the age group of 20-70 years. This finding aligns with observations from several prior studies that reported a higher prevalence of vitamin B12 deficiency among females compared to males [7, 16, 17]. Factors such as inadequate dietary intake, overcooking of food, poor absorption, and low socioeconomic status likely contribute to vitamin B12 insufficiency in our community. Additionally, specific life stages and conditions, such as pregnancy and lactation, may further impact vitamin B12 levels in females, making them more susceptible to deficiency [18].

To better understand the vitamin B12 status of our patients, we stratified megaloblastic anemia patients based on their vitamin B12 levels into five groups: very low, low, borderline low, normal, and high levels. Among the 95 patients, 34.7% had very low levels, 14.7% had low levels, 14.7% had borderline low levels, 31.6% had normal levels, and 4.3% had high levels of B12. The p-value for this distribution was 0.000, indicating a significant variation in vitamin B12 levels among the patients. Our results align with those of Nizamani et al. (2014), who categorized vitamin B12-deficient patients into similar groups [7]. Interestingly, 4.3% of patients in our study exhibited high vitamin B12 levels, which corresponds to the findings of a study where 5.6% of patients had elevated vitamin B12 levels due to underlying myeloproliferative disorders [19].

In our study, we made efforts to rule out diseases such as hypothyroidism, cardiac diseases, gastrointestinal disorders, pulmonary tuberculosis, and liver disease, all of which can elevate vitamin B12 levels. By focusing on a specific patient group with megaloblastic anemia, the study provides targeted insights that are highly relevant for clinical practice in diagnosing and managing this condition. The study adds valuable data to the limited research on diagnostic markers for megaloblastic anemia in Pakistan, contributing to the understanding of vitamin B12 deficiency in this population. Additionally, we attempted to ascertain whether patients had recently taken B12 supplementation. However, many patients were not well educated and belonged to a low socioeconomic class, making it challenging to obtain this information. For patients presenting with symptoms of B12 deficiency, investigations may reveal elevated serum cobalamin levels, accompanied by an increase in MMA levels. This functional cobalamin deficiency may arise due to increased binding of vitamin B12 to haptocorrin, leading to a reduction in the delivery of B12 to peripheral cells [20]. Relying solely on serum B12 assays can result in up to a 50% misdiagnosis rate [16]. Serum cobalamin levels may fall within the normal range in patients with clinical signs of cobalamin deficiency. Therefore, tests such as serum Holo-TC and MMA levels are appropriate next steps to rule out vitamin B12 deficiency.

Low Holo-TC levels indicate vitamin B12 deficiency. Our study results demonstrated that Holo-TC levels were low in all patients (n = 94), except for one patient who had normal Holo-TC levels. High MMA levels indicate vitamin B12 deficiency. Present study results demonstrated that 93 (97.9%) patients had elevated MMA levels, while only two patients showed normal MMA levels. Our study showed a sensitivity of 98.9% for Holo-TC, with a specificity of 50.00%. The PPV was 98.91%, and the NPV was 50%. Conversely, the sensitivity of vitamin B12 was 63%, with a specificity of 50%, a PPV of 98.33%, and an NPV of 2.85%. The diagnostic accuracy of HoloTC was observed to be 97.8%, while that of vitamin B12 was 63.15%. Our study results are consistent with those of previous studies [11, 13, 21], all of which found Holo-TC to be a more sensitive marker for detecting B12 deficiency than serum B12 levels.

Our study revealed a high prevalence of vitamin B12 deficiency in younger populations, with 36.8% falling in the 20-30-year age group. This finding is in line with observations by a study that reported a 45% prevalence of B12 deficiency in the 21-40 age group [15]. Additionally, our study showed that non-vegetarians were more likely to experience vitamin B12 deficiency, with 84% of patients being non-vegetarian. These results align with a study where the frequency of vitamin B12 deficiency in vegetarians was 78.5%, compared to 85% in non-vegetarians [18].

The current study was constrained by a small sample size due to financial limitations. Future research should consider larger sample sizes to enhance the diagnostic specificity of Holo-TC. Additionally, the availability of MMA testing is limited, and both cobalamin and MMA levels can fluctuate over time in ambulatory care settings, which may not predict cobalamin-responsive diseases. Nonetheless, Holo-TC appears to be a superior test for identifying vitamin B12 deficiency, and this study contributes to the limited research on diagnostic markers for megaloblastic anemia in Pakistan. To facilitate its integration into routine diagnostic workflows, clinicians could adopt a stepwise approach: initially testing patients with symptoms of vitamin B12 deficiency using Holo-TC as a primary screening tool, followed by MMA testing for borderline or ambiguous cases. Regular monitoring of high-risk populations, such as the elderly and those with malabsorption syndromes, using Holo-TC could further improve early detection and treatment outcomes. These recommendations align with the growing body of evidence supporting Holo-TC as a more sensitive marker than total B12.

## Conclusions

In conclusion, our study found that Holo-TC is a more sensitive marker than serum cobalamin levels for detecting vitamin B12 deficiency, with a sensitivity of 98.9%, specificity of 50.00%, PPV of 98.90%, and NPV of 50%. Conversely, vitamin B12 exhibited lower sensitivity (63%), specificity (50%), PPV (98.33%), and NPV (2.85%). The diagnostic accuracy of HoloTC was 97.8%, while that of vitamin B12 was 63.15%. Our study emphasizes the importance of Holo-TC as an effective tool for identifying vitamin B12 deficiency and suggests its use as a primary diagnostic parameter. Further research with larger sample sizes and diverse populations is recommended to validate these findings and to explore the integration of Holo-TC testing into routine clinical workflows.
